# Genomic heterogeneity affects the response to Daylight Saving Time

**DOI:** 10.1038/s41598-021-94459-z

**Published:** 2021-07-20

**Authors:** Jonathan Tyler, Yu Fang, Cathy Goldstein, Daniel Forger, Srijan Sen, Margit Burmeister

**Affiliations:** 1 Department of Mathematics, University of Michigan, Ann Arbor, MI 48109 USA; 2 Division of Pediatric Hematology/Oncology, Department of Pediatrics, University of Michigan, Ann Arbor, MI 48109 USA; 3 Michigan Neuroscience Institute, University of Michigan, Ann Arbor, MI 48109 USA; 4 Department of Computational Medicine and Bioinformatics, University of Michigan, 109 Zina Pitcher Place, Ann Arbor, MI 5061 BSRB48109 USA; 5 Department of Neurology, University of Michigan, Ann Arbor, MI 48109 USA

**Keywords:** Behavioural genetics, Psychology and behaviour

## Abstract

Circadian clocks control the timing of many physiological events in the 24-h day. When individuals undergo an abrupt external shift (e.g., change in work schedule or travel across multiple time zones), circadian clocks become misaligned with the new time and may take several days to adjust. Chronic circadian misalignment, e.g., as a result of shift work, has been shown to lead to several physical and mental health problems. Despite the serious health implications of circadian misalignment, relatively little is known about how genetic variation affects an individual’s ability to entrain to abrupt external changes. Accordingly, we used the one-hour advance from the onset of daylight saving time (DST) as a natural experiment to comprehensively study how individual heterogeneity affects the shift of sleep/wake cycles in response to an abrupt external time change. We found that individuals genetically predisposed to a morning tendency adjusted to the advance in a few days, while genetically predisposed evening-inclined individuals had not shifted. Observing differential effects by genetic disposition after a one-hour advance underscores the importance of heterogeneity in adaptation to external schedule shifts. These genetic differences may affect how individuals adjust to jet lag or shift work as well.

## Introduction

Circadian (about a day) clocks are fundamental processes in most organisms that time many molecular, physiological, and behavioral events across the 24-h day^1^, such as regulating hormone release^2^, body temperature^3^, metabolism^4^, and sleep/wake patterns^5^. In humans, these clocks are synchronized (i.e., *entrained*) to the environment primarily through the external light–dark cycle^6^. The Suprachiasmatic Nucleus (SCN), found in the hypothalamus, receives light input and acts as the master pacemaker that synchronizes peripheral clocks according to the external light cues^7^. Although the SCN pacemaker itself adjusts to changes in the environment relatively quickly, peripheral clocks can take longer to adjust^7^. Thus, outputs of the circadian clock in an individual, e.g., the sleep/wake cycle, do not instantaneously entrain to abrupt external shifts^7^. Importantly, the magnitude of desynchronization between the central and peripheral clocks that results from abrupt external schedule changes demonstrates significant interindividual variability^8–12^.

Temporary desynchronization is commonly associated with travel across several time zones causing the annoying effect of “jet lag”. When an individual travels across several time zones, the relationship between clock time and the environment is maintained while the individual’s circadian phase is desynchronized from the environment. Another common desynchronization is due to the clock change from daylight saving time (DST). In contrast to jet lag, in DST, the clock time arbitrarily changes while the environment does not, leading to a desynchronization between circadian phase and clock time but not circadian phase and environment. This artificial change in clock time leads to impaired sleep at night and excessive daytime sleepiness as well as other dramatic consequences such as increased automotive accidents^13–15^ and heart attacks^16, 17^.

Previous studies have used the DST transition as a unique opportunity to investigate how individuals adjust to external time changes^18–23^. However, these previous studies were limited in power due to small sample sizes and often reported contradictory results^23^. Moreover, these analyses relied on subjective self-reporting, thereby limiting the traits that could be captured and often misrepresenting personal sleep information^24^. Finally, these contradictory results regarding the impact of DST may be because studies failed to account for circadian tendency, often evaluated as chronotype^25^, and interindividual heterogeneity in sleep behavior.

Now, however, genome-wide association studies (GWAS) provide objective measures that describe predisposition to certain diseases and phenotypes given individual genetic variation^26^. In particular, recent work has revealed genetic associations with sleep midpoint, as objectively measured from accelerometers, rather than biased self-reported sleep information^24^. This validated and objective genetic sleep midpoint association provides a surrogate measure to characterize an individual's genomic predisposition to morningness or eveningness, specifically as it relates to the sleep/wake cycle.

Here, we use the one-hour advance from the onset of daylight saving time as a natural experiment to comprehensively study how heterogeneity affects an individual’s ability to entrain to an abrupt external time change. In total, from a cohort of 831 medical interns^27^ with genotype information, we analyze thousands of sleep events the week before and the week after DST onset in 2019. After analysis, we see that morning-inclined individuals adjust more rapidly to the time shift than evening-inclined. Moreover, we find that social jet lag^28^ is exaggerated in evening-inclined individuals the week after DST. Ultimately, our results provide a more comprehensive evaluation of how genetic predispositions affect how the sleep/wake cycle patterns shift in response to an external change. Since DST is only a one-hour advance, observing differential effects by genetic disposition underscores the importance of heterogeneity in more extreme cases such as shift work. Additionally, our results further establish the utility of the polygenic score as a surrogate measure of phenotypes. Finally, understanding how subtle phenotypic differences affect shifting patterns will help inform individuals on how to prepare for abrupt external shifts (e.g., DST, changes in work or school schedules, transmeridian travel, etc.), and ultimately, advance our understanding of circadian misalignment.

## Results

### Sleep midpoint in the morning group is significantly earlier than that in the evening group

**Figure 1:**
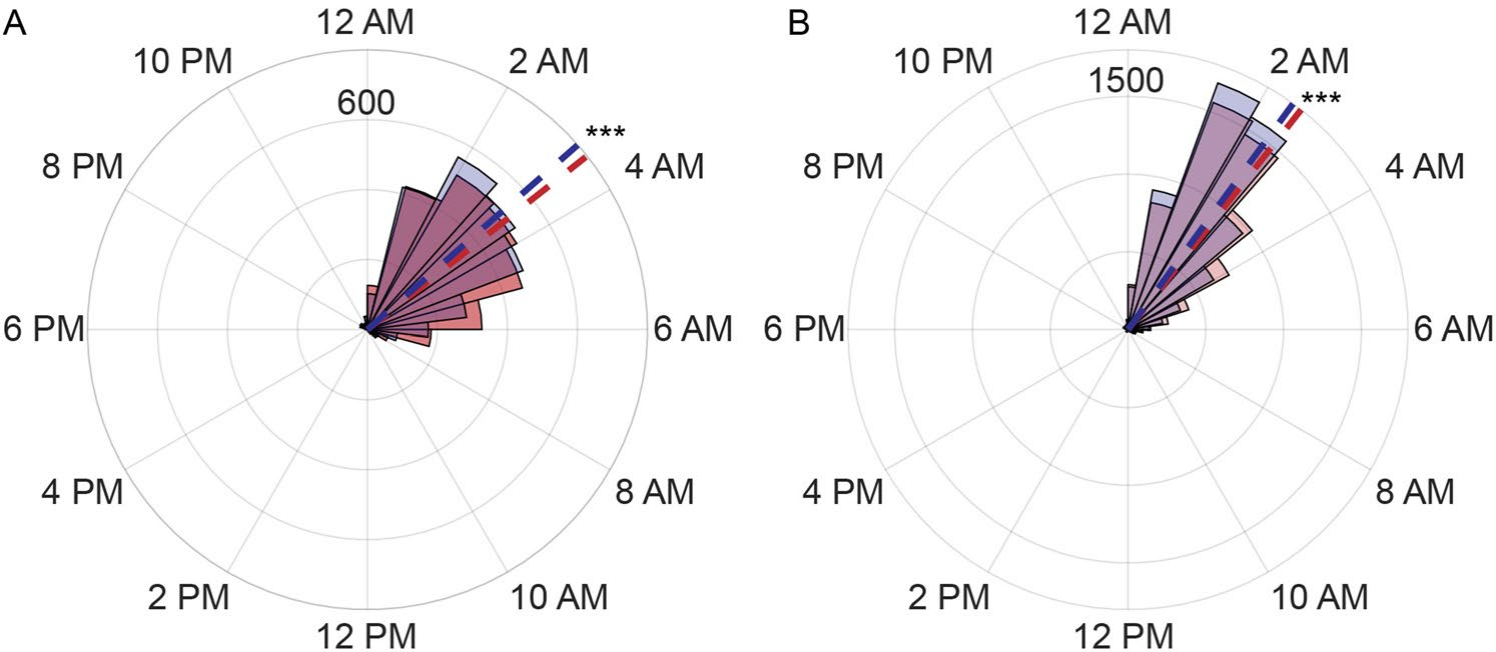
Sleep midpoints are significantly different in the two groups. The distribution of sleep midpoints in the morning (blue shade) group is earlier than that of the evening (red) group on free nights (**A**) and work nights (**B**). The mean sleep midpoints are plotted as dashed lines. On free nights (**A**), the mean sleep midpoint of the morning group is 3:15 AM (n = 2771 sleep events), and the mean sleep midpoint of the evening group is 3:26 AM (n = 2754 sleep events, F-statistic = 12.83, P = 3.43e−4, Parametric Watson-Williams multisample test for equal means). On work nights (**B**), the mean sleep midpoint of the morning group is 2:25 AM (n = 7296), and the mean sleep midpoint of the evening group is 2:33 AM (n = 7227 sleep events, F-statistic = 20.15, P = 7.2e−6, Parametric Watson-Williams multisample test for equal means).

We used the Objective Sleep Midpoint polygenic score (OSM PGS), calculated using a genome-wide chronotype genetic analysis of 85,670 individuals^24^, as an objective measure for chronotype rather than subjective self report. Specifically, we calculated the OSM PGS with the methods described previously^29, 30^ for all subjects with genetic information (N = 831). As a higher OSM PGS corresponds to a later sleep midpoint, we divided all PGS scores into three quantiles and characterized the top quantile as an evening group and the bottom quantile as a morning group. In Fig. 1, using all the sleep events up to the onset of DST, we plot the distribution of sleep midpoints for the morning and evening groups both on free (e.g., Friday-Saturday, Fig. 1A) and work (e.g., Sunday-Thursday, Fig. 1B) nights. As expected, the morning group had significantly earlier sleep midpoints on both free and work nights, and the evening group displayed later sleep midpoints (Fig. 1).

### Evening population sleeps significantly less on work nights following DST relative to the week before

**Figure 2:**
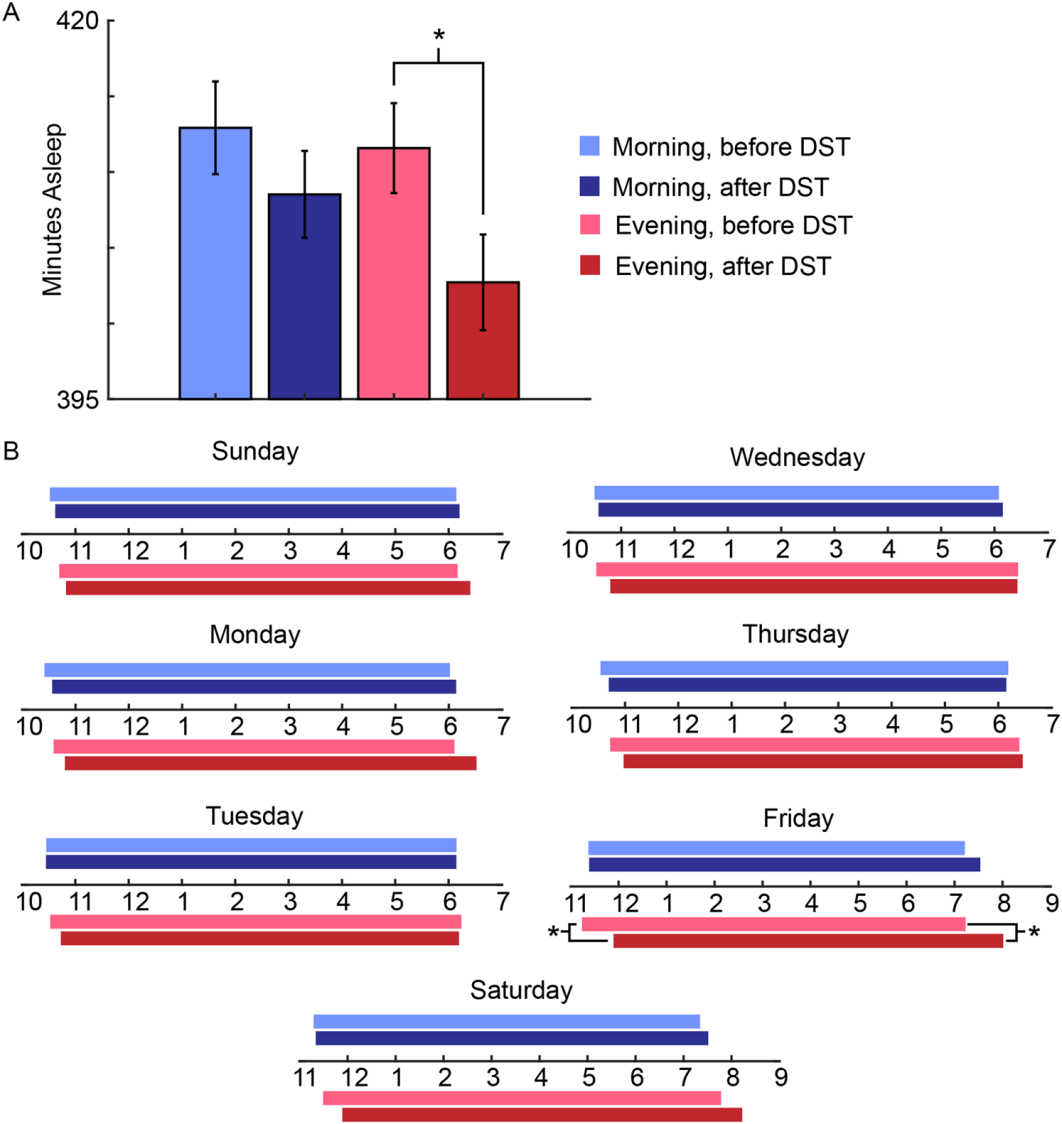
Analysis of Sleep Events before and after DST starts. (**A**) The mean asleep time on work nights (i.e., Sunday-Thursday nights) for the morning population is consistent across the week before DST (412.9 ± 3 min, n = 676 sleep events) and the week after DST (408.5 ± 2.9 min, n = 671 sleep events). The mean asleep time on work nights for the evening population decreases from 411.6 ± 2.97 min (n = 651 sleep events) to 402.7 ± 3.16 min the week after DST (n = 602 sleep events, T-statistic = − 2.0451, P = 0.041). (**B**) Mean sleep profiles of the morning (blue) and evening (red) populations for the night of the week before (lighter shade) and after DST (darker shade). For statistical analysis on circular data, we use the Parametric Watson-Williams multisample test for equal means (F-statistic = 5.11 and P = 0.025 for sleep onset and F-statistic = 6.73 and P = 0.0102 for sleep offset). See Table 1 for the sample sizes in each group.

For the week before and after DST onset, we computed the mean time asleep, in minutes, for nighttime sleep events on Sunday-Thursday (work) nights for both the morning and evening groups (Fig. 2A). We saw a marginal decrease in the average time asleep in the morning population (mean 412.9 ± 3 min before and 408.5 ± 2.9 min after DST, Fig. 2A). In contrast, the mean time asleep decreased in the evening population (from 411.6 ± 2.97 min before to 402.7 ± 3.16 min the week after, P = 0.041, Fig. 2A). Altogether, the group with the later circadian tendency could not successfully shift their sleep schedules to align with the one-hour shift forward.

### Morning population shifts to the new time in three days while the evening population has not shifted 1 week after DST

For both populations, we computed the mean sleep onset and offset times for each day of the week before and after DST (Fig. 2B). In the morning population, sleep onset times are delayed on the Sunday and Monday nights after DST (Fig. 2B). However, after Monday night, the sleep profiles of the morning population are nearly identical across the week before and after DST. Moreover, on the freer nights (e.g., Friday and Saturday), the morning population sleep profiles continue to remain nearly identical across the two weeks.

The evening type population also exhibited sleep onset times that are delayed on the Sunday and Monday nights after DST. Furthermore, unlike the morning population, the evening population continued to exhibit later sleep onset and offset times the week after DST compared with the week before DST (Fig. 2B). Importantly, the sleep onset times display a more dramatic delay relative to the week before than the offset times, which are likely fixed due to work schedule constraints. Because work schedule constraints are less on weekends, the differences in sleep profiles are more dramatic on the Friday and Saturday night after DST. In fact, on Friday, there is a significant difference in the sleep onset time of around 45 min and in the sleep offset time of around 40 min (Fig. 2B). Therefore, even one week after DST, the evening population has not shifted to the new external time.

### Social jet lag significantly increases in the evening population the week after DST

**Figure 3:**
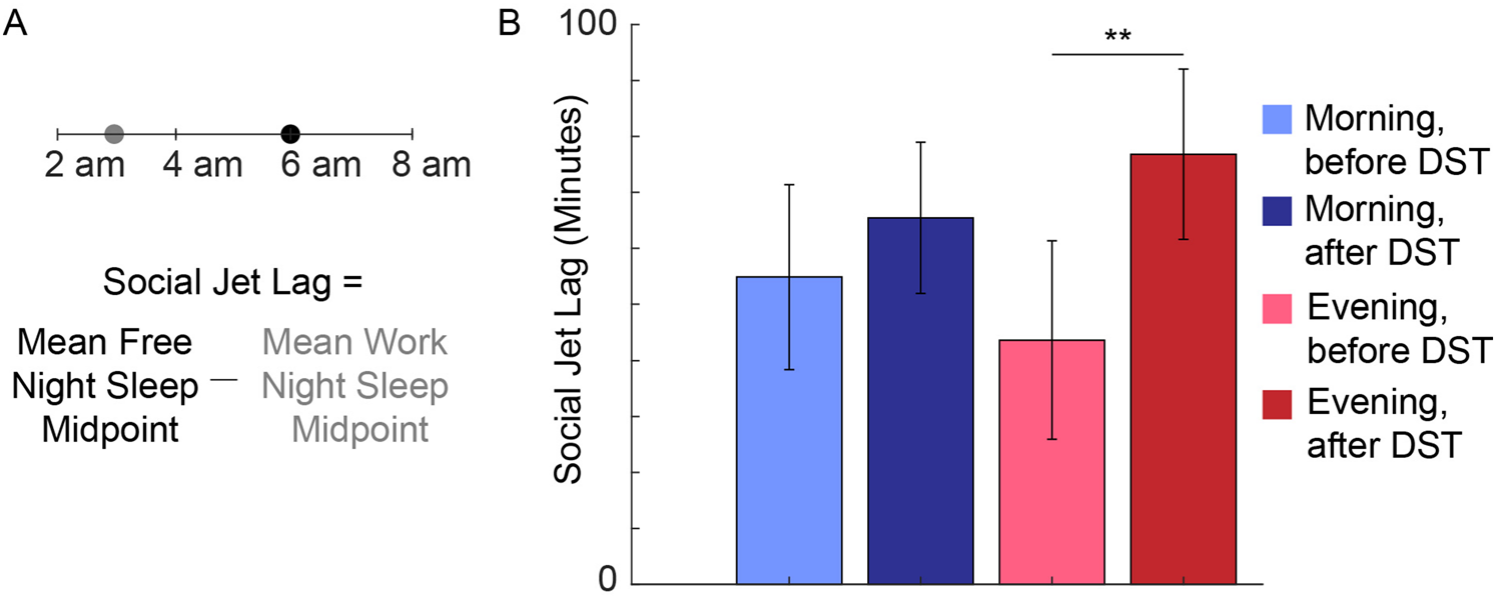
Social jet lag is significantly higher in the evening population after DST. (**A**) Schematic of the social jet lag calculation. For each individual with at least one work night sleep event and one free night sleep event, we calculate the mean sleep midpoint for the workweek and the mean sleep midpoint for the free nights. Then, social jet lag is defined as the difference between the mean free night sleep midpoint and the mean work night sleep midpoint. (**B**) For the morning population, social jet lag is consistent across the week before DST (n = 133 subjects) and the week after DST (n = 141 subjects). For the evening population, social jet lag significantly decreased (T-statistic = − 2.83, P = 0.0051) the week after DST (n = 134 subjects) compared with the week before DST (n = 118 subjects).

Finally, we assessed social jet lag, the difference between the sleep midpoint on workdays and free days^25, 28^, the weeks before and after DST in each population (Fig. 3A). Specifically, we grouped the sleep midpoints of Monday-Thursday nights before and after DST as the work-night sleep midpoints. Similarly, we group the sleep midpoints on Friday and Saturday nights as the free-night sleep midpoints.

Social jet lag is significantly increased in the evening population (from 44 to 77 min, Fig. 3B) after DST. Thus, even though the evening population continues to have a later sleep onset each night of the week following DST (Fig. 2B), the difference in midpoint from the work nights to the following Friday is significantly more pronounced. In contrast, we see only a small increase in the social jet lag of the morning population (55 to 65 min, Fig. 3B) after DST, providing further evidence that the morning population has successfully shifted to the time change the weekend following DST.

## Discussion

Here, we performed a comprehensive analysis of how individual heterogeneity affects an individual’s ability to adjust to an abrupt shift. We used the Objective Sleep Midpoint polygenic score^24, 29, 30^ to characterize individual heterogeneity in sleep/wake cycles. In particular, we labeled morning-inclined individuals as those with an OSM PGS in the bottom third of all scores, and we labeled evening-inclined individuals as those with an OSM PGS in the top third of all scores. This characterization revealed significant differences in sleep midpoints on both free and work nights, similar in magnitude to other reports^24^. Thus, we used the OSM PGS as a surrogate marker for chronotype.

In summary, we saw dramatic differences in adaptation to the one hour DST time shift between individuals genetically predisposed to a morning versus evening circadian inclination. First, the evening group exhibited lower average time asleep in the week following DST relative to the week before, corroborating previous work which reported a decrease in sleep duration the week after DST relative to the previous week^21^. The lower average time asleep is particularly concerning in a population of medical interns, for which it has previously been shown that they are already sleep-deprived^31^. Additional lack of sleep the week after DST in the evening group may increase medical errors or exaggerate depressive symptoms, more likely in evening individuals^32^.

Further analysis revealed that both groups exhibited the expected delay in sleep onset and offset for the Sunday and Monday nights after DST onset. Thus, even though there may be a build-up of sleep pressure due to less sleep the Saturday night of DST onset, it is not enough to result in an instantaneous shift, contrary to a previous report^20^. Rather, after Monday, the sleep timing of the morning population realigned to the sleep schedule before DST. Given that the experimental setting was everyday life with several external and environmental signals, we cannot completely rule out the possibility that this realignment is due to masking rather than entrainment. Masking refers to the signals that affect the rhythmic output directly rather than the core pacemaker and only last through the duration of the signal^33^. On the other hand, entrainment refers to a shift in the core pacemaker that allows for future anticipation of events even after removal of the signal. Here, since the sleep profiles are consistent pre- and post-DST after Monday, we conclude that the morning group entrained to the new time.

In contrast to the morning population, the evening population sleep profiles continued to exhibit a delay in sleep onset for the rest of the workweek. Assessment of sleep timing on free days demonstrated that the evening population had not shifted to the time change one week after DST onset. That is, on free nights a week after DST (i.e., Friday and Saturday), the evening population showed a drastic delay in both sleep onset and offset relative to the week before DST. Thus, the workweek schedule masks the magnitude to which the sleep/wake cycles in the evening population are misaligned to the new time. Moreover, given fixed wake-up times due to the interns’ rigid work requirements, the evening population had a significant increase in social jet lag the week after DST onset relative to the week before. This result further supports the difficulty evening individuals have in phase advancing to realign with external time in response to the DST time shift.

Morning individuals relative to the evening-inclined individuals may shift so quickly because these individuals have a more rapid build-up of sleep loss, and thus, after an advance, they can shift their bedtimes earlier^34^. On the other hand, the variability in how the two groups respond to light at certain clock and circadian phase times may explain the disparity in the ability to entrain to a new time. For instance, a previous study showed that morning chronotype individuals receive significantly more light at early morning clock times than evening chronotypes but receive significantly less in the evening clock hours^35^. Even though morning individuals receive less light in the evening, they receive significantly more light input late in the clock phase (0–4 h before melatonin onset) than the evening individuals^35^. Moreover, Phillips et. al showed that there is significant interindividual variability in sensitivity to evening light^36^. Given these results, morning individuals may better mitigate higher light levels later in their respective biological phase. Coupled with the result that morning individuals receive more light in the early clock time, morning individuals may shift quicker to DST onset because of more light input in the morning, which is diminished by DST onset, as well as mitigating higher light levels in the evening, also a result of DST onset.

The significant increase in social jet lag highlights the desynchronization between the independent homeostatic sleep/wake and internal circadian sleep rhythms^37^ in the evening population. The sleep/wake cycle, as seen in the work nights, does not display as dramatic of a shift because of the increase in sleep pressure. However, the internal biological clock drive, seen in the phase of the free nights, continues to be delayed one week after DST in the evening population. As the desynchronization among free and work nights remains relatively constant in the morning population, the more extreme desynchronization in the sleep timing among free and work nights in the evening population underscores that the magnitude of desynchronization between peripheral and central clocks displays significant interindividual variability. The evening population’s decreased ability to mitigate abrupt shifts may result in a higher likelihood of adverse health events or medical errors.

We acknowledge that our study is limited by the fact that all participants are from a cohort of medical Interns. While this limits generalizability, it also provides the advantage that the age range is limited, avoiding having to account for age effects on circadian behavior. Another limitation is that we do not consider the geographical location of the subjects, investigated previously^23, 38^. Dawn times vary widely around the spring equinox, and thus, around the spring DST transition with a differential effect across latitudes^23^. Therefore, individuals at certain latitudes may mitigate the shift better than at other latitudes because of stronger sunlight at earlier dawn times.

Altogether, our results demonstrate the power of using polygenic scores in differentiating individual genomic predisposition to morningness or eveningness and how these characterizations reveal interindividual variability in shifting to external changes. Observing significant differential effects after a one-hour external shift emphasizes that genetic variation may play an important role in mitigating more extreme shifts, and thus, how individuals respond to chronic misalignment due to shift work and the extent to which individuals suffer mental and physical health problems as a result.

## Methods

### Participants

The Intern Health Study (IHS) is a multi-site cohort study that follows physicians across several institutions in their first year of residency. The intern participants entered residency in 2018 and were contacted via email 2–3 months before residency onset regarding details of the study and provided informed written consent to participate. Interns that consented were given a $25 gift certificate upon completion of a baseline survey and another $25 gift certificate after completing a follow-up survey^27, 39^. Both the Institutional Review Boards at the University of Michigan and participating institutions approved the study, and all work was carried out in accordance with these approvals and all relevant regulations.

### Sleep data capture

All subjects were invited to wear a Fitbit Charge 2™, a consumer fitness tracker that tracks sleep patterns, heart rate, and physical activity. The Fitbit Charge 2™ uses an accelerometer and photoplethysmography sensor as well as proprietary algorithms to quantify sleep. Although the Fitbit Charge 2™ is not an FDA cleared medical device, it demonstrates a 0.96 sensitivity (accuracy to detect sleep) and 0.61 specificity (accuracy to detect wake) when compared to laboratory polysomnography in healthy adults^40^. Furthermore, although summary sleep metrics demonstrated that the Fitbit Charge 2™ overestimated PSG total sleep time (TST) by 9 ± 24 min and underestimated PSG sleep onset latency (SOL) by 4 ± 9 min, it was similar to PSG in determining wake after sleep onset (WASO)^40^.

The Fitbit reports start and end time, total minutes asleep, total minutes in bed, and other metrics for each sleep event. We used these values directly from the Fitbit when possible for performing our analysis. We accounted for the circular nature of sleep data by using circular statistic functions from the Circular Statistics Toolbox in Matlab^41^. For example, we saved all sleep start and end times and normalized them to a value between 0 and 2π (0 corresponds to midnight and π corresponds to noon) and computed the mean sleep midpoints using the circular mean function. We then transformed the mean back to an hour by dividing by 2π and multiplying by 24 to project it to the 24-h day.

### Sleep event classification and analysis

#### Nighttime vs. daytime classification

We characterized each sleep event as a nighttime sleep event if it started between the hours of 6 pm and 6 am the following morning. Otherwise, the sleep event was characterized as a daytime sleep event. Furthermore, we classified each sleep event by the day of the week on which it started. For example, a sleep event that started after 6 pm on Sunday but before 6 am the following Monday was classified as a Sunday nighttime sleep event even if it technically started on Monday.

#### Weekday classification

To assess the ability to shift to the external time change, we further refined the data set, following the convention introduced in previous studies examining sleep changes after DST^18, 20^. In particular, we characterized sleep events as being the week before DST if they were nighttime sleep events from Saturday, March 2nd, to Friday, March 8th. Similarly, we characterized sleep events as being the week after DST onset if they were nighttime sleep events from Sunday, March 10th, to Saturday, March 16th. In this way, we guaranteed, as much as possible, a uniform representation of sleep events starting each night of the week. Table 1 reports a breakdown of the number of sleep events each night of the week both before and after DST onset.

**Table 1:** Number of weekday sleep events before and after DST.

Weekday	Sleep events before DST	Morning PGS before DST	Evening PGS before DST	Sleep events after DST	Morning PGS after DST	Evening PGS after DST
Sunday	463 (423)	148 (130)	127 (119)	481 (420)	150 (134)	136 (113)
Monday	485 (440)	154 (137)	143 (127)	461 (424)	148 (134)	130 (122)
Tuesday	479 (440)	147 (134)	142 (131)	474 (434)	150 (138)	135 (126)
Wednesday	496 (460)	149 (140)	155 (144)	474 (433)	148 (137)	128 (118)
Thursday	471 (428)	147 (137)	143 (131)	478 (431)	144 (130)	133 (125)
Friday	473 (436)	152 (138)	135 (126)	481 (426)	144 (133)	147 (126)
Saturday	444 (397)	130 (111)	124 (115)	470 (419)	141 (125)	141 (130)
Total	3311 (3024)	1027 (927)	969 (893)	3319 (2987)	1025 (931)	950 (860)

Here, we list the total number of nighttime sleep events the weeks before and after DST onset starting on the respective day of the week (columns 2 and 5). Furthermore, we list the number of sleep events of the morning population (before DST, column 3 and after DST, column 6) and the evening population (columns 4 and 7). The values listed in parentheses are the number of sleep events that are greater than four hours.

#### PGS classification

We then subdivided the sleep events both before and after DST onset based on whether the subject was labeled as morning- or evening-inclined. In Table 1, we breakdown the number of sleep events in the morning and evening groups for the weeks before and after DST. In the end, we analyzed 1027 (969) sleep events the week before DST and 1025 (950) sleep events the week after DST for the morning group and 969 (893) before and 950 (860) after for the evening group (Table 1). These sleep events were distributed uniformly across the seven weeknights in each population (Table 1).

To account for the effect of shift schedules, we only used sleep events from subjects that had sleep events in both weeks on the same day of the week. I.e., for the Monday night sleep analysis, we only took sleep events from subjects that had a sleep event the Monday before and the Monday after DST. Furthermore, we rejected any sleep event that was less than four hours long to remove any bias due to interruptions in a normal sleep event (e.g., an interruption because the subject was called into work in the middle of the night). In this way, we removed, as much as possible, any bias introduced into the data from shift changes. Altogether, after removing sleep events that were less than four hours, we lost about ten percent of the sleep events. See the parentheses values in Table 1 for the number of sleep events that were longer than four hours, and thus, the total number of sleep events used in the analysis.

#### Mean sleep onset and end times computation

We computed the mean sleep onset time for each group on each day of the week by taking all nighttime sleep events longer than four hours and transforming each to an angle by multiplying by 2π and dividing by 24. We then took the circular mean using the circ_mean function in the Circular Statistics Toolbox. We then transformed the mean angle back by dividing by 2π and multiplying by 24 to project back to hours. We repeated this for each day of the week and each group both before and after DST onset. We performed the same procedure to compute the mean sleep end time.

#### Social jet lag analysis

To analyze social jet lag, we first found all subjects that had at least one work night (Monday-Thursday) sleep event and one free night (Friday-Saturday) sleep event the week before DST. We then calculated the social jet lag for the individual as the mean sleep midpoint on free nights minus the mean sleep midpoint on work nights. We performed the same procedure for the week after DST. Note that we did not require subjects to be in both the week before and the week after, just one or the other.

### Genotyping and PGS calculation

We collected DNA from 2018 cohort subjects (n = 1,624) using DNA Genotek Oragene Mailable Tube (OGR-500)^42^ through the mail. DNA was extracted and genotyped on Illumina Infinium CoreExome—24 with v.1.3 Chips, containing 595,427 single nucleotide polymorphisms (SNPs). We then implemented a quality check of genotype data with PLINK v.1.9 (www.cog-genomics.org/plink/1.9/)^43^. Samples with call rate < 99% (n = 18) or with a sex mismatch between genotype data and reported data (n = 4) were excluded. Duplicated samples (n = 3) with a lower call rate were excluded. We only included SNPs on autosomal chromosomes, with call rate ≥ 98% (after sample removal) and minor allele frequency (MAF) ≥ 0.005. A total of 331,077 SNPs and 1599 samples were considered for further analysis. Of the 1599 samples, we analyzed 831 that had corresponding wearable data. The samples contained mixed ancestries to increase sample size and power.

To calculate the PGS of objective sleep midpoint, we used the GWAS summary statistics of accelerometer-based sleep midpoint from UK Biobank containing 85,670 samples^24^. We used PRSice v.2^29^ to calculate OSM-PGS for our intern subjects. The OSM-PGS was calculated using all the 225,466 variants genotyped in our sample (with MAF ≥ 0.1 and outside the major histocompatibility complex (MHC) region) that overlap with summary statistic data from the OSM GWAS (i.e., p-value thresholds = 1). The OSM-PGS was then calculated with a weighted additive model:

$$PGS =\Sigma S \times G$$

where S is the MDD GWAS summary statistic effect size for the effect allele and G = 0, 1, 2 is the number of effect alleles observed. The OSM-PGS was then mean-centred and scaled to 1 SD.

### Statistical analyses

We performed a two-sample t-test at a significance level of 95% to assess whether mean time asleep was significantly different before and after DST in the two groups independently. For the circular variables such as the sleep midpoints, sleep start, and sleep end times, we assessed significance by performing a Parametric Watson-Williams multi-sample significance test for unequal means at a significance level of 95%. For the social jet lag analysis, we assessed significance using a two-sample t-test at a significance level of 95%.

## Data Availability

The de-identified data from Intern Health Study is available through the Psychiatric Genomics Consortium (PGC): https://www.med.unc.edu/pgc/shared-methods. PGC phase 2–UK Biobank–23andMe MDD GWAS meta-analysis summary statistics: https://www.nature.com/articles/s41593-018-0326-7#data-availability.
